# Post-Myocardial Infarction Ventricular Septal Defect in the Wall of a Basal Ventricular Aneurysm

**Published:** 2018-01

**Authors:** Ali Hosseinsabet, Jamshid Bagheri

**Affiliations:** *Tehran Heart Center, Tehran University of Medical Sciences, Tehran, Iran.*

**Keywords:** Myocardial infarction, Heart septal defects* ventricular, Aneurysm

A 59-year-old man with 3-vessel disease, candidated for coronary artery bypass grafting surgery, was referred to our echocardiography ward. One month prior to his referral to us, the patient had suffered inferior myocardial infarction, for which he underwent selective coronary angiography in another center. The procedure revealed significant stenosis in all 3 major epicardial coronary arteries. Our physical examinations revealed systolic murmurs at the left sternal border and apex. Electrocardiography revealed Q-wave and T-wave inversion in the inferior leads. Transthoracic echocardiography demonstrated severe left ventricular enlargement with a reduced systolic function (ejection fraction = 45%), severe right ventricular enlargement with moderate-to-severe systolic dysfunction, biatrial enlargement, severe functional mitral regurgitation, and moderate-to-severe tricuspid regurgitation. Additionally, this evaluation showed a huge aneurysm in the basal segment of the inferior and inferoseptal wall of the left ventricle, protruding into the right ventricle. There was also a ventricular septal defect (10 mm) in the wall of this aneurysm with a left-to-right shunt. Transesophageal echocardiography confirmed these findings. The patient was referred for revascularization, valvular repair, and closure of the ventricular septal defect. The hemodynamics of these patients usually deteriorate, and the mortality rate of medical treatment is high. Surgical or percutaneous closure of post-myocardial infarction ventricular septal defects reduces the mortality rate; the rate, however, is significant. Our patient’s hemodynamics were relatively stable, which was unusual. The presence of a ventricular septal defect is an uncommon complication after myocardial infarction, and the presence of a ventricular septal defect in the wall of an aneurysm is even a rarer finding. Accordingly, in the evaluation of patients with basal aneurysms, the presence of this associated lesion should be kept in mind.

**Figure 1:**
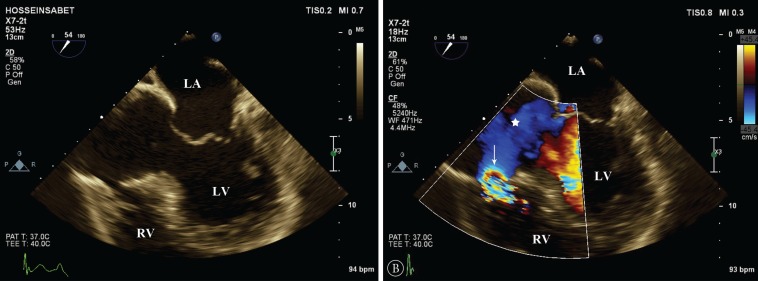
Huge aneurysm (*) in the basal segment of the inferoseptal wall of the left ventricle, protruding into the right ventricle alongside a ventricular septal defect (arrow) in the wall of this aneurysm without (A) and with color Doppler study (B) in transesophageal echocardiography.

LA, Left atrium; LV, Left ventricle; RV, Right ventricle

***To watch the following videos, please refer to the relevant URLs:***

Video 1. Huge aneurysm in the basal segment of the inferoseptal wall of the left ventricle, protruding into the right ventricle with a ventricular septal defect in the wall of this aneurysm in transesophageal echocardiography.

http://jthc.tums.ac.ir/index.php/jthc/article/view/790/635

Video 2. Huge aneurysm in the basal segment of the inferoseptal wall of the left ventricle, protruding into the right ventricle alongside a ventricular septal defect in the wall of this aneurysm with color Doppler study in transesophageal echocardiography.

http://jthc.tums.ac.ir/index.php/jthc/article/view/790/636

## Notes:

***This paper should be cited as: ***Hosseinsabet A, Bagheri J. Post-Myocardial Infarction Ventricular Septal Defect in the Wall of a Basal Ventricular Aneurysm. J Teh Univ Heart Ctr 2018;13(1)38-39.

